# Chromatin conformation signatures of cellular differentiation

**DOI:** 10.1186/gb-2009-10-4-r37

**Published:** 2009-04-19

**Authors:** James Fraser, Mathieu Rousseau, Solomon Shenker, Maria A Ferraiuolo, Yoshihide Hayashizaki, Mathieu Blanchette, Josée Dostie

**Affiliations:** 1 Department of Biochemistry and McGill Cancer Center, McGill University, 3655 Promenade Sir-William-Osler, Montréal, H3G1Y6, Canada; 2 McGill Centre for Bioinformatics, McGill University, 3775 University, Montréal, H3A 2B4, Canada; 3 RIKEN Omics Science Center, RIKEN Yokohama Institute, 1-7-22 Suehiro-cho Tsurumi-ku, Yokohama, 230-0045, Japan

## Abstract

One of the major genomics challenges is to better understand how correct gene expression is orchestrated. Recent studies have shown how spatial chromatin organization is critical in the regulation of gene expression. Here, we developed a suite of computer programs to identify chromatin conformation signatures with 5C technology . We identified dynamic *HoxA *cluster chromatin conformation signatures associated with cellular differentiation. Genome-wide chromatin conformation signature identification might uniquely identify disease-associated states and represent an entirely novel class of human disease biomarkers.

## Abstract 2

A suite of computer programs to identify genome-wide chromatin conformation signatures with 5C technology is reported.

## Rationale

Cell specialization is the defining hallmark of metazoans and results from differentiation of precursor cells. Differentiation is characterized by growth arrest of proliferating cells followed by expression of specific phenotypic traits. This process is essential throughout development and for adult tissue maintenance. For example, improper cellular differentiation in adult tissues can lead to human diseases such as leukemia [1,2]. For this reason, identifying mechanisms involved in differentiation is not only essential to understand biology, but also to develop effective strategies for prevention, diagnosis and treatment of cancer. Suzuki *et al*. recently defined the underlying transcription network of differentiation in the THP-1 leukemia cell line [3]. Using several powerful genomics approaches, this study challenges the traditional views that transcriptional activators acting as master regulators mediate differentiation. Instead, differentiation is shown to require the concerted up- and down-regulation of numerous transcription factors. This study provides the first integrated picture of the interplay between transcription factors, proximal promoter activity, and RNA transcripts required for differentiation of human leukemia cells.

**Figure 1:**
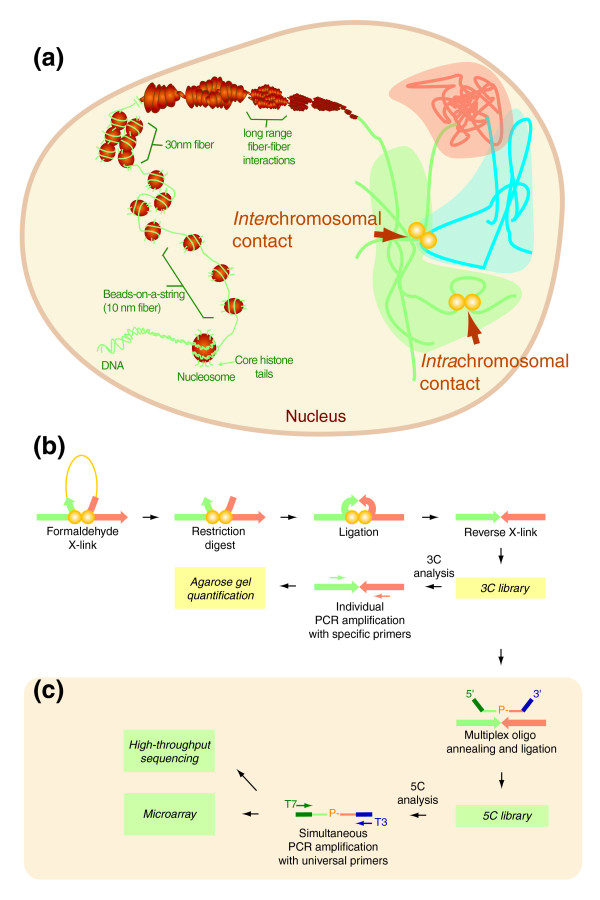
Capturing spatial chromatin organization *in vivo *with 3C/5C technologies. **(a) **Current model of genome organization in the interphase nucleus. The diagram illustrates multiple levels of chromatin folding from the primary structural unit consisting of genomic DNA bound to nucleosomes (10 nm fiber; left). Secondary organization levels involve formation of 30 nm fibers through nucleosome-nucleosome interactions, and binding of individual fibers is believed to form tertiary structures (top). Folded chromatin occupies 'chromosome territories' represented by green, blue or orange shaded areas (right). Yellow circles indicate physical DNA contacts within (intra) or between (inter) chromosomes. **(b) **Schematic representation of 3C technology. 3C measures *in vivo *cross-linked DNA contacts at high resolution using individual PCR amplification and agarose gel detection. Interacting DNA segments located in *cis *is shown as an example to illustrate the 3C approach. *Cis*-interacting DNA fragments are represented by green and orange arrows and separated by a given genomic region (yellow line; left). Yellow circles represent cross-linked proteins. DNA segments are illustrated by arrows to highlight 'head-to-head' ligation configurations quantified by 3C. **(c) **Schematic representation of the 5C technology. 5C measures DNA contacts from 3C libraries using multiplex ligation-mediated amplification and microarray or high-throughput DNA sequencing. Genomic homology regions of 5C primers are shown in green and orange, and universal primer sequences are colored dark green or blue.

Although extremely powerful, several observations indicate that implementation of new technologies will be required to gain a full appreciation of how cells differentiate. First, gene expression is controlled by a complex array of regulatory DNA elements. Each gene may be controlled by multiple elements and each element may control multiple genes [4]. Second, the functional organization of genes and elements is not linear along chromosomes. For example, a given element may regulate distant genes or genes located on other chromosomes without affecting the ones adjacent to it [4,5]. Third, gene regulation is known to involve both local and long-range chromatin structure changes [6,7]. Although the role of histone and DNA modifications is increasingly well described, relatively little is known about the function of spatial chromatin organization in the regulation of genes. Interestingly, recent studies show that control DNA elements can mediate long-range *cis *or *trans *regulation by physically interacting with target genes [8-10]. These studies indicate that genomes are organized into dynamic three-dimensional networks of physical DNA contacts essential for proper gene expression (Figure 1a). Therefore, mapping the functional (physical) connectivity of genomes is essential to fully identify the mechanisms involved in differentiation, and might provide important diagnostic and prognostic signatures of human diseases.

Physical contacts between DNA segments can be measured with the 'chromosome conformation capture' (3C) technologies [11,12]. The 3C approach (Figure 1b) uses formaldehyde to covalently link chromatin segments *in vivo*. Cross-linked chromatin is then digested with a restriction enzyme and ligated under conditions promoting intermolecular ligation of cross-linked segments. Cross-links are finally reversed by proteinase K digestion and DNA extraction to generate a '3C library'. 3C libraries contain pair-wise ligation products, where the amount of each product is inversely proportional to the original three-dimensional distance separating these regions. These libraries are conventionally analyzed by semi-quantitative PCR amplification of individual 'head-to-head' ligation junctions and agarose gel detection (for details, see [12]). 3C was first used to show that long-range interactions are essential for gene expression in several important mammalian genomic domains. For example, it was demonstrated that the locus control region of the beta-globin locus specifically interacts with actively transcribed genes but not with silent genes [13-16]. These contacts were required for gene expression and mediated by the hematopoietic transcription factors GATA-1 and co-factor FOG-1 [15].

3C technology has been widely adopted for small-scale analysis of chromatin organization at high-resolution [17-24]. However, this approach is technically tedious and not convenient for large-scale studies. Genome-scale conformation studies can be performed quantitatively using the 3C-carbon copy (5C) technology (Figure 1c) [16,25]. The 5C approach combines 3C with the highly multiplexed ligation-mediated-amplification technique to simultaneously detect up to millions of 3C ligation junctions. During 5C, multiple 5C primers corresponding to predicted 'head-to-head' 3C junctions are first annealed in a multiplex setting to a 3C library. Annealed primers are then ligated onto 3C contacts to generate a '5C library'. Resulting libraries contain 5C products corresponding to 3C junctions where the amount of each product is proportional to their original abundance in 3C libraries. 5C libraries are finally amplified by PCR in a single step with universal primers corresponding to common 5C primer tails. These libraries can be analyzed on custom microarrays or by high-throughput DNA sequencing [16]. Although 5C technology is an ideal discovery tool and particularly well suited to map functional interaction networks, this approach is not yet widely adopted partly due to the lack of available resources.

In this study, we used the THP-1 leukemia differentiation system characterized by Suzuki *et al*. [3] to identify chromatin conformation signatures (CCSs) associated with the transcription network of cellular differentiation. To this end, we mapped physical interaction networks with the 3C/5C technologies in the transcriptionally regulated *HoxA *cluster and in a silent gene desert region. The *HoxA *genes were selected for their pivotal roles in human biology and health. Importantly, the *HoxA *cluster encodes 2 oncogenes, *HoxA9 *and *HoxA10*, which are over expressed in THP-1 cells. This genomic region plays an important role in promoting cellular proliferation of leukemia cells and *HoxA *CCS identification should, therefore, help understand the mechanisms involved in regulating these genes.

Using 3C, we found that repression of *HoxA9*, *10*, *11 *and *13 *expression is associated with formation of distinct contacts between the genes and with an overall increase in chromatin packaging. Chromatin remodeling was specific to transcriptionally regulated domains since no changes were observed in the gene desert region. We developed a suite of computer programs to assist in 5C experimental design and data analysis and for spatial modeling of 5C results. We used these tools to generate large-scale, high-resolution maps of both genomic regions during differentiation. 5C analysis recapitulated 3C results and identified new chromatin interactions involving the transcriptionally regulated *HoxA *region. Three-dimensional modeling provided the first predicted conformations of a transcriptionally active and repressed *HoxA *gene cluster based on 5C data. Importantly, these models identify CCSs of human leukemia, which may represent an entirely novel class of human disease biomarker. 5C research tools are now publicly available on our 5C resource website (see Materials and methods).

## Results and discussion

### Spatial chromatin remodeling accompanies *HoxA *gene repression during cellular differentiation

**Figure 2:**
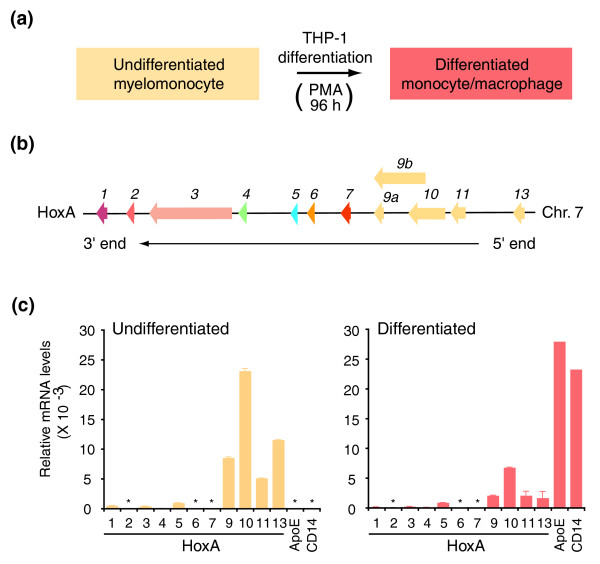
5' end *HoxA *genes are repressed during cellular differentiation. **(a) **Cellular differentiation system used in this study. The human myelomonocytic cell line THP1 was stimulated with PMA to cease proliferation and induce differentiation into mature monocytes/macrophages. **(b) **Linear schematic representation of the human *HoxA *gene cluster on chromosome 7. Genes are represented by left facing arrows to indicate direction of transcription. Cluster is presented in a 3' (*HoxA1*) to 5' (*HoxA13*) orientation. Same family members are labeled with identical color. Paralogue groups (*1*-*13*) are identified above each gene. **(c) **Quantitative real-time PCR analysis of *HoxA *genes during cellular differentiation. Steady-state mRNA levels in undifferentiated (left) and differentiated cells (right) were normalized relative to actin. CD14 and ApoE expression levels were measured to verify cellular differentiation. Number below each histogram bar identifies paralogue group. Asterisks indicate mRNA expression below quantitative real-time PCR detection levels. Each histogram value is the average of at least three PCRs and error bars represent the standard deviation.

We mapped physical interaction networks of the *HoxA *cluster and of a control gene desert region in the THP-1 differentiation system characterized by Suzuki *et al*. [3]. THP-1 are myelomonocytic cells derived from an infant male with acute myeloid leukemia. These cells terminally differentiate into mature monocytes/macrophages following stimulation with phorbol myristate acetate (PMA; Figure 2a) [26-28]. THP-1 cells express the *MLL*-*AF9 *fusion oncogene originating from the translocation t(9;11)(p22;q23) between the mixed-lineage leukemia (*MLL*) and *AF9 *genes [29,30]. MLL gene rearrangements are frequently found in both therapy-related and infantile leukemia, and promote cellular proliferation by inducing aberrant expression of oncogenes, including *HoxA9 *and *A10 *[31-35].

*Hox *genes encode transcription factors of the homeobox superfamily [36]. In mammals, there are 39 *Hox *genes organized into 4 genomic clusters of 13 paralogue groups. The *HoxA*, *B*, *C*, and *D *clusters are each located on different chromosomes. For example, the *HoxA *cluster is located on human chromosome 7 and encodes 11 evolutionarily conserved genes (Figure 2b). Undifferentiated THP-1 cells are known to express high levels of 5' end *HoxA *genes, which are repressed following PMA-induced differentiation [3]. We first verified that *HoxA *genes were regulated in our samples by measuring steady-state mRNA levels with quantitative real-time PCR (Figure 2c). As expected, we found that *HoxA9*, *10*, *11 *and *13 *were highly expressed in undifferentiated THP-1 compared to the other paralogues (Figure 2c, left). Expression of these genes was significantly reduced following differentiation (Figure 2c, right), whereas the macrophage-specific ApoE and CD14 markers were induced in mature monocytes/macrophages. These results indicate that *HoxA *genes are correctly regulated under our experimental conditions. RT-PCR primer sequences used in this analysis are presented in Additional data file 1.

**Figure 3:**
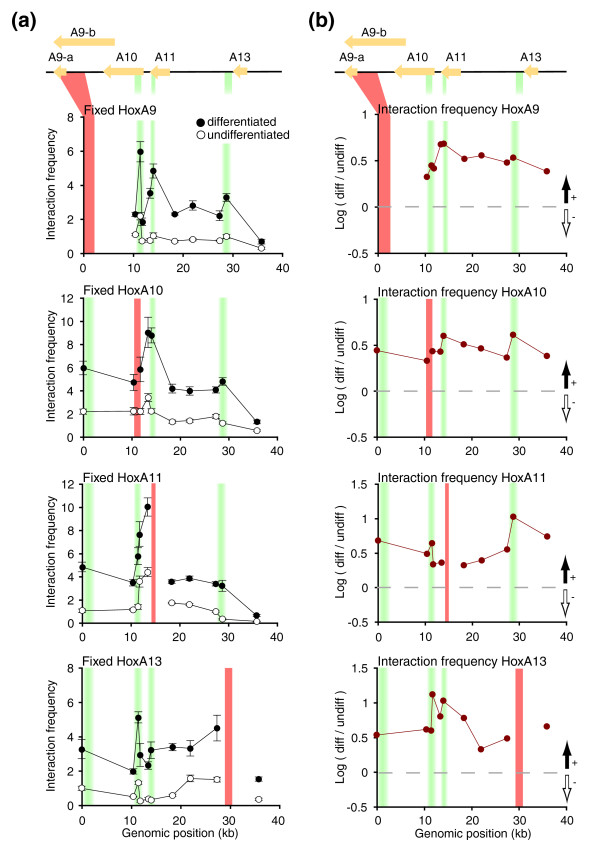
Extensive spatial chromatin remodeling accompanies 5' *HoxA *gene repression during cellular differentiation. **(a) **Conventional 3C analysis of transcriptionally regulated *HoxA *genes. Chromatin contacts between the *HoxA9*, *A10*, *A11*, or *A13 *genes and surrounding genomic domain were measured in undifferentiated and differentiated cells. The y-axis indicates normalized interaction frequency; the x-axis shows genomic position relative to start of domain characterized. The genomic domain is shown to scale above the graphs, and is as described in Figure 2b. Solid orange vertical lines identify the position of the 'fixed' 3C region analyzed in each graph. Shaded green vertical lines highlight the position of putative DNA looping contacts. Each data point is the average of at least three PCRs. Error bars represent the standard error of the mean. **(b) **Chromatin contact changes during cellular differentiation. 3C interactions between the *HoxA9*, *A10*, *A11*, or *A13 *genes and surrounding genomic domain presented in (a) were compared in both cellular states by calculating fold differences (log ratio differentiated/undifferentiated). Areas above and below horizontal dashed lines represent increased and reduced interactions in differentiated cells, respectively (black and white vertical arrows). The genomic domain is shown to scale above the graphs as in (a). Interaction frequencies represent the average of at least three PCRs and error bars represent the standard error of the mean.

*Hox *genes are master regulators of development and play pivotal roles during adult tissue differentiation. During development, the expression of *Hox *genes is regulated both spatially and temporally in an order that is colinear with their organization along chromosomes [37-39]. This colinearity has fascinated biologists for over 25 years and strongly suggests that chromatin structure plays an important role in their regulation. We first used the conventional 3C method to determine whether *HoxA *gene regulation is accompanied by changes in spatial chromatin architecture. 3C libraries from undifferentiated and differentiated THP-1 cells, and a control library prepared from bacterial artificial chromosome (BAC) clones were generated as described in Materials and methods. These libraries were used to characterize chromatin contacts within the transcriptionally regulated 5' end *HoxA *region (Figure 3a, b, top). In undifferentiated cells, the *HoxA9 *promoter region was found to interact frequently with neighboring fragments ('Fixed *HoxA9*' in Figure 3a). Additionally, the interaction frequency (IF) did not rapidly decrease with increasing genomic distance. In contrast, *HoxA9 *repression in differentiated cells was accompanied by formation of very strong looping contacts and by overall increased interaction frequency. Interestingly, looping fragments contained other down-regulated genes, suggesting that *HoxA *repression involves increased chromatin packaging mediated by the specific clustering of co-regulated genes.

To determine whether all or only specific genes interact with each other when repressed, we mapped the interaction profile of each looping fragment in both cellular states ('Fixed *HoxA10*, *11*, *13*' in Figure 3a). Similarly to *HoxA9*, *HoxA10*, *11*, and *13 *interacted frequently with neighboring fragments in undifferentiated and differentiated cells. Interaction frequency did not rapidly decrease with increasing genomic distance in undifferentiated cells. In fact, weaker but similar interaction profiles were observed in both cellular states, which is consistent with the partial gene repression measured in our samples (Figure 2c). We found that all repressed genes formed strong looping contacts with each other following differentiation and that silencing was accompanied by overall increased interaction frequency (Figure 3b). Looping contact intensities were likely underrepresented since *HoxA9*-*13 *gene expression was reduced rather than completely silenced in our samples (Figure 2c). Therefore, *HoxA *gene repression during cellular differentiation involves overall increased chromatin packaging driven, at least in part, by looping and clustering of co-repressed genes.

**Figure 4:**
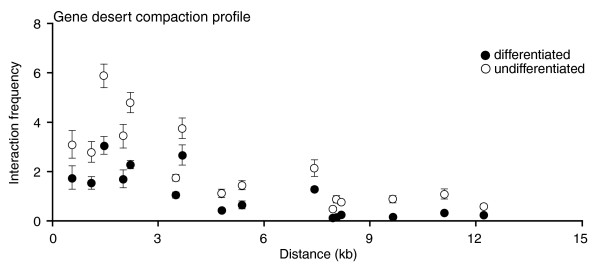
The chromatin compaction of a gene desert control region does not significantly change during cellular differentiation. The y-axis indicates interaction frequency and the x-axis shows genomic distance between interacting fragments. The average log ratio of corresponding contacts in undifferentiated and differentiated cells from this dataset was used to normalize the *HoxA *3C datasets shown in Figure 3a. Interaction frequencies represent the average of at least three PCRs and error bars represent the standard error of the mean.

Direct quantitative comparison of IFs between cellular states was achieved by measuring contacts in a gene desert region as previously described (Figure 4) [12]. The gene desert characterized in this study is thought to be transcriptionally silent and should, therefore, remain unchanged following cellular differentiation. Accordingly, we found similar chromatin compaction profiles in both cell states where IFs decreased with increasing genomic distance. This result is consistent with a linear random-coil chromatin fiber devoid of long-range looping contacts. 3C primer sequences used in this analysis are presented in Additional data file 2.

Together, these results demonstrate that the spatial chromatin organization of the *HoxA *cluster is dynamic and depends upon transcription activity. Low-resolution *in situ *hybridization analysis of the *HoxB *and *D *clusters during mouse embryonic stem cell differentiation previously demonstrated that temporal *Hox *induction is accompanied by changes in spatial chromatin architecture [40-42]. For example, retinoic acid *HoxB *gene induction was shown to induce global decondensation and physical exclusion of the cluster from its chromosome territory. This 'looping out' mechanism was conserved in the *HoxD *cluster, suggesting that similar chromatin remodeling mechanisms regulate different *Hox *clusters. Interestingly, the *Drosophila *homeotic bithorax complex was recently found to be organized into higher-order chromosome structures mediated by the polycomb response elements [43]. In our preliminary 3C analysis we demonstrate that the corresponding human *HoxA *genes are also organized into looping contacts when transcriptionally repressed. These results strongly suggest that an evolutionarily conserved structural mechanism regulates the expression of *Hox *genes. Comprehensive mapping of the gene clusters will be required both to define the mechanism(s) regulating Hox expression and identify conserved Hox CCSs of cellular differentiation.

### 5C array analysis of *HoxA *spatial chromatin remodeling during cellular differentiation

We characterized 3C libraries with 5C technology to generate high-resolution maps of the entire *HoxA *cluster and control gene desert region during THP-1 differentiation. 5C analysis has been hampered by the lack of publicly available research tools. For this reason, we developed several computer programs to assist in experimental design, data analysis and result interpretation. First, we generated '5CPrimer' to design forward and reverse 5C primers directly from any given genomic domain. This program selects primers based on sequence complexity, length, and melting temperatures, and excludes sequences homologous to DNA repeats. This program is extensively described in the Materials and methods and an example of 5CPrimer output is presented in Additional data file 3.

We used 5CPrimer to design the *HoxA *and gene desert oligonucleotides used in this study (Additional data file 3). 5C libraries were generated with 58 5C primers using the cellular and control 3C libraries characterized above as templates (Figure S1a in Additional data file 4). Libraries were produced with alternating forward and reverse primers corresponding to consecutive restriction fragments along each region, and contained up to 841 different contacts. These contacts include 441 interactions within the *HoxA *cluster, 64 in the gene desert region, and 336 inter-chromosomal genomic contacts. This experimental design yields the maximum interaction coverage achievable per 5C library (50%), and generates a matrix of interactions throughout both genomic domains. To verify that multiplexed 5C libraries contained quantitative 3C contact 'carbon copies', we measured the levels of four 5C products regulated during THP-1 differentiation (Figure S1b, c in Additional data file 4; Figure 3a, b). 5C ligation products were measured individually with internal primers as previously described [16]. We found that 5C libraries closely recapitulated the 3C interaction profiles in both cellular states, indicating quantitative detection of chromatin contacts in our 5C libraries. 5C internal primer sequences are shown in Additional data file 5.

We analyzed the 5C libraries generated above using custom microarrays. To facilitate 5C array design, we developed the '5CArray' program. This program uses output files of the 5CPrimer algorithm and can design custom 5C arrays from any genomic region. A detailed description of this program is presented in Materials and methods. We used 5CArray to design the custom 5C microarrays used in this study. 5C libraries were hybridized onto arrays as described previously, and normalized IFs were calculated with the 'IF Calculator' program. We developed IF Calculator to automate IF calculation and exclusion of signals close to background (see Materials and methods). We first verified that 5C array results recapitulate 3C analysis by comparing the 3C and 5C chromatin interaction profiles of four different cluster regions regulated during THP-1 differentiation (Additional data file 6). We found that 5C array results recapitulated the overall interaction profiles generated by conventional 3C. However, some variations were observed, which may be explained by differences in the dynamic range of each approach as previously reported [16].

**Figure 5:**
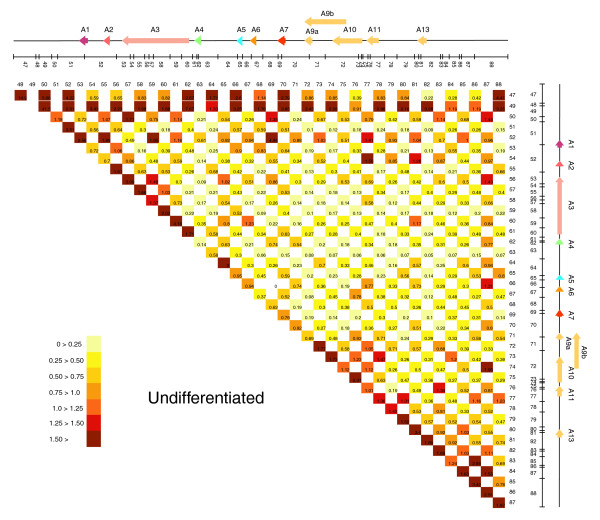
5C array analysis of chromatin conformation changes in the *HoxA *cluster during cellular differentiation. *HoxA *chromatin contacts in undifferentiated cells are presented as a two-dimensional heat map. Pair-wise interaction frequencies between restriction fragments were detected by 5C and measured on custom microarrays. A linear diagram of the *HoxA *gene cluster is presented at the top and right borders and is as described in Figure 2b. A predicted *Bgl*II restriction pattern is illustrated below the *HoxA *diagram and is to scale. Restriction fragments were identified from left to right by the numbers indicated below each line. Intersecting column and row numbers identify DNA contact. Values within each square represent interaction frequencies and are color-coded. The color scale is shown in the bottom left inserts, with pale yellow to brown indicating very weak to strongest contacts. Interaction frequencies are the average of at least three array technical repeats. Note: primer 48 was included during large-scale 5C library production but was excluded from our analysis because of homology to repetitive sequences.

**Figure 6:**
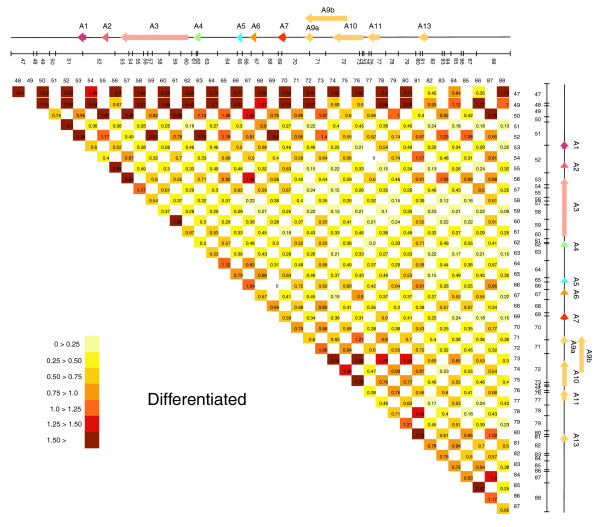
5C array analysis of chromatin conformation changes in the *HoxA *cluster during cellular differentiation. *HoxA *chromatin contacts in differentiated cells are presented as a two-dimensional heat map as described in Figure 5.

To help visualize spatial chromatin architecture changes between cellular states, we represented the complete *HoxA *5C interaction maps as two-dimensional heat maps where the color of each square is a measure of pair-wise IFs (Figure 5 & Figure 6). Several changes can be observed from these maps. First, THP-1 differentiation is associated with overall increased chromatin packaging (compare overall IFs from each map). Second, gain of contacts throughout the cluster in differentiated cells is accompanied by decreased IFs between neighbors (compare IFs along diagonals in each map). This result is consistent with the formation of looping interactions and with a linear detection of DNA contacts in our experimental system. Third, the 3' end of the cluster (fragments 47-50) interacts very strongly with the entire *HoxA *region in both samples, suggesting that this region might be located at the center of the model. Fourth, chromatin remodeling mostly involved the 3' end (fragments 47-50) and the transcriptionally regulated 5' end (fragments 71-75) of the cluster.

**Figure 7:**
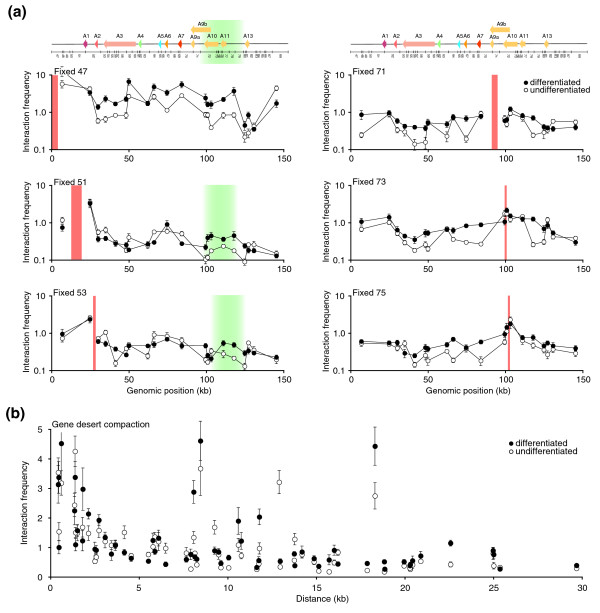
Extensive *HoxA *spatial chromatin remodeling during cellular differentiation involves the transcriptionally regulated 5' end region. **(a) **5C chromatin interaction profiles with the greatest differences between undifferentiated and differentiated states were extracted from 5C datasets. The normalized interaction frequency is plotted logarithmically on the y-axis to emphasize differences between cellular states. The x-axis shows genomic position relative to the start of the domain analyzed. The linear *HoxA *cluster diagram and predicted *Bgl*II restriction pattern are shown to scale above the graphs, and are as described in Figures 2b, 5 & 6. Solid orange vertical lines identify the position of 'fixed' 5C interaction profiles presented in each graph. Shaded green vertical lines highlight position of putative 3'-5' looping regions. Each data point is the average of at least three array interaction frequencies. Error bars represent the standard error of the mean. **(b) **5C chromatin compaction of a gene desert control region does not change during differentiation. The y-axis indicates interaction frequency and the x-axis shows genomic distance between interacting fragments. The average log ratio of corresponding contacts in undifferentiated and differentiated cells from this dataset was used to normalize *HoxA *5C datasets shown in Figures 5 & 6 and in (a). Interaction frequencies represent the average of at least three array interaction frequencies and error bars represent the standard error of the mean.

To identify the most regulated chromatin contacts, we then compared the individual interaction profiles of each restriction fragment in both cell states (Figure 7a). We found that interaction between the 3' end and the entire *HoxA *cluster greatly increased following differentiation (Fixed 47 in Figure 7a). We also found that the transcriptionally regulated region interacted more frequently throughout the cluster in differentiated cells (Fixed 71, 73, 75 in Figure 7a). Interestingly, fragments containing the *HoxA1 *and *A2 *genes interacted more frequently with this region after differentiation (Fixed 51, 53 in Figure 7a; green highlight). These results suggest that transcription repression of 5' end genes induces formation of long-range DNA contacts between the ends of the cluster. Because the maximum interaction coverage achievable per 5C library is 50%, looping contacts were not well defined in this experiment (compare Figures 7a and 3a). However, higher resolution can be obtained by combining complementary 5C datasets or by performing 5C on 3C libraries generated with frequent cutters (for example, *Dpn*II).

In this experiment, we also used the control gene desert region to normalize IFs between datasets and to determine whether extensive chromatin remodeling was specific to transcriptionally regulated domains (Figure 7b). As observed by 3C, similar chromatin compaction profiles were found in both cell states. IFs rapidly decreased with increasing genomic distance, which is consistent with a linear chromatin fiber devoid of long-range looping contacts. These results suggest that extensive chromatin remodeling occurs preferentially in transcriptionally regulated regions during cellular differentiation. Therefore, CCSs might be valuable predictive signatures of gene expression and may represent an entirely novel class of human disease biomarker.

### Computer modeling of *HoxA *spatial chromatin architecture

Two-dimensional analysis of 5C interaction maps identified several *HoxA *chromatin contacts regulated during differentiation. However, this preliminary analysis revealed an important feature of 5C detection of chromatin remodeling in that regulation involves both gain and loss of contacts throughout regulated domains (compare Figure 5 and Figure 6). Because two-dimensional data analysis mainly identifies prominent changes in DNA contacts, this approach does not fully integrate spatial chromatin regulation and information is lost. For this reason, we developed the '5C3D' modeling program, which uses the 5C datasets to generate a representation of the average three-dimensional conformation based on IFs. 5C3D posits that relative IFs are inversely proportional to the physical distance between DNA segments *in vivo*. Starting from a random three-dimensional structure, 5C3D moves points iteratively to improve the fit to the physical distances estimated from the IFs (see Materials and methods for details). No model was found to match exactly all pairwise distances, although the deviations were small for all pairs of points. This result is likely due to IF variability that may originate from experimental error, very low or high signals, or from experimental design. For example, 5C datasets generated from cell populations contain averaged IFs derived from various cell cycle states, which can introduce noise in models. For these reasons, 5C3D generates averaged structural models rather then true individual *in vivo *structures. Nevertheless, the model generated by this modeling program, while not providing a 'true' structure for the chromosome's conformation, still represents a valuable CCS identification tool.

**Figure 8:**
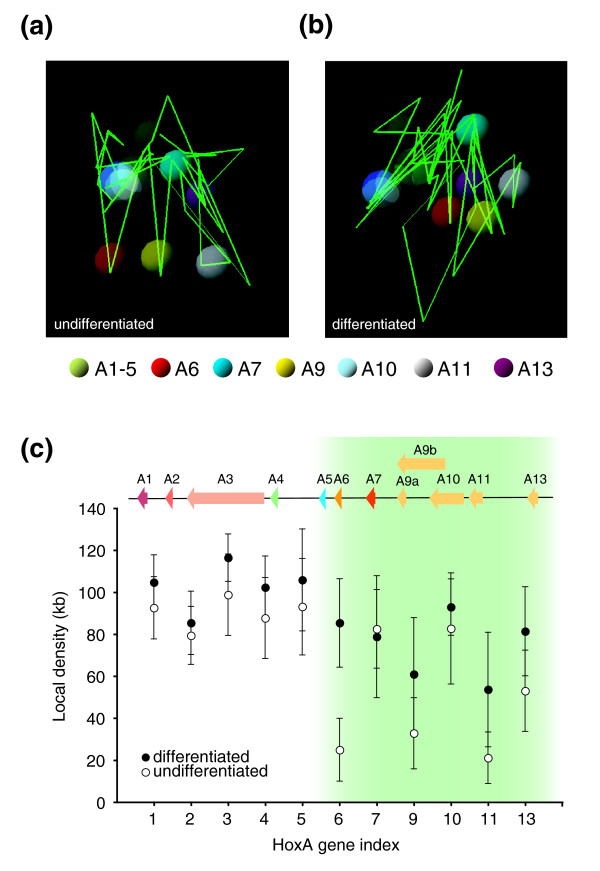
Three-dimensional models of the human *HoxA *cluster during cellular differentiation. 5C array datasets from **(a) **undifferentiated and **(b) **differentiated samples were used to predict models of the *HoxA *cluster with the 5C3D program. Green lines represent genomic DNA and vertices define boundaries between consecutive restriction fragments. Colored spheres represent transcription start sites of *HoxA *genes as described in the legend. **(c) **Increased local genomic density surrounding 5' *HoxA *transcription start sites accompanies cellular differentiation. The y-axis indicates local genomic density and *HoxA *paralogue groups are identified on the x-axis. A linear schematic representation of the *HoxA *cluster is shown at the top, and green shading highlights the region of greatest density change. Error bars represent standard deviations.

We used 5C3D to predict three-dimensional models of the *HoxA *cluster in undifferentiated and differentiated cells (Figure 8a, b). In these models, the overall spatial chromatin density of the *HoxA *cluster increased following differentiation. This result is consistent with increased IFs observed in 5C datasets and, importantly, correlates with transcription repression of 5' end genes. For example, we found that transcriptionally silent 3' end *HoxA *genes (*A1*-*5*) were spatially clustered in undifferentiated cells and that this organization did not significantly change following differentiation. However, the position of transcriptionally regulated genes was significantly altered between cell states. In undifferentiated cells, *HoxA9*, *11 *and *13 *are expressed and looped away from the cluster. In contrast, these genes were pulled back towards the cluster following transcription repression in differentiated cells. The relative position of *HoxA10 *did not significantly change following differentiation where, accordingly, it remained the most highly expressed 5' end gene (Figure 2c). We also found that the position of a region containing *HoxA6 *was significantly altered following differentiation. Since this gene is transcriptionally silent in both conditions, this result suggests that physical exclusion of genes from the cluster is not sufficient for transcription induction.

Visual identification of chromatin conformation changes from three-dimensional models can be challenging particularly when 5C3D outputs are sensitive to noise in IFs. To help robustly identify differences between models, we developed the 'Microcosm' program. Microcosm uses 5C datasets to calculate local chromatin densities within any given genomic environment, which are then represented graphically. This program minimizes error from model variability and statistically interprets differences by using multiple predicted conformations based on a set of pair-specific models of noise in IFs (see Materials and methods for details). Although Microcosm measures only density and not identity of surrounding DNA, this program is nonetheless useful to visualize conformational changes as manageable two-dimensional 'molecular imprints'.

We used Microcosm to estimate local chromatin densities around *HoxA *genes in both cellular states (Figure 8c). We found that transcriptionally silent 3' end *HoxA *genes (*A1*-*5*) reside in comparable local density environments (see Additional data file 7 for calculated *p*-values). These environments did not change significantly following differentiation, which is consistent with the predicted 5C3D models (Figure 8a, b). In contrast, local densities around *HoxA9*, *11*, and *13 *increased significantly upon transcription repression to levels approaching those of the silent 3' end *HoxA *genes. Also consistent with predicted 5C3D models, the local density of *HoxA10 *was comparable in both cell states, whereas the environment of transcriptionally silent *HoxA6 *dramatically changed following differentiation. The reason for chromatin remodeling at the transcriptionally silent *HoxA6 *gene region remains unknown. However, its position between transcriptionally silent and regulated domains might identify it as a molecular hinge during formation of contacts between the ends of the cluster following cellular differentiation.

Nothing is known about the mechanisms involved in the establishment and/or maintenance of *HoxA *DNA contacts during differentiation. However, the CAGE (cap analysis of gene expression) and chromatin immunoprecipitation (ChIP)-chip datasets generated by Suzuki *et al*. under both cellular conditions correlated well with our findings [3]. For example, CAGE, which quantitatively identifies transcription start sites at high resolution, specifically detected transcription start sites upstream of the *HoxA9*, *10*, *11 *and *13 *genes in undifferentiated cells. Consistent with our results, these transcription start sites were significantly repressed following differentiation. Moreover, transcription repression of 5' end genes was specifically correlated with reduced acetylated histone (H3K9Ac) and RNA polymerase II association, which are two markers of active transcription. Complete mapping of chromatin modifications in the cluster should help understand the role of DNA contacts in *HoxA *gene regulation throughout cellular differentiation and in human leukemia cells.

### Comparison to similar software

We developed a suite of publicly available 5C computer programs to promote mapping of functional interaction networks in any non-specialized molecular biology laboratory. No software similar to '5CArray', 'IF Calculator', '5C3D', or 'Microcosm' existed prior to this study. A rudimentary program used to predict 5C primer sequences was previously developed in collaboration with NimbleGen Systems Inc. [16] but was not usable by non-specialists. The original script was written in Perl, was command line only, and required the installation of several additional packages to function. The '5CPrimer' computer program presented in this study was written in C as a command line tool, but a web interface was created for easy access and use of all features for users of all abilities. 5CPrimer does not require additional packages to work, but is designed to make use of the RepeatMasker, if installed, to eliminate repetitive sequences that can potentially cause problems. The output files from the 5CPrimer program are used as the input for the 5CArray program.

## Conclusions

In this study, we identified CCSs associated with transcription networks of cellular differentiation in a human leukemia cell line. The dynamic *HoxA *CCSs reported here are reminiscent of the three-dimensional structures recently described in the *D. melanogaster *homeotic bithorax complex [44]. Therefore, our results suggest that an evolutionarily conserved mechanism based on chromatin architecture regulates the expression of *Hox *genes. However, CCS mapping of each *Hox *cluster in other human differentiation systems will be required to verify evolutionary conservation of these signatures. The role of chromatin contacts in the regulation of Hox genes is still unknown and it will be particularly interesting to determine whether chromatin architecture is required for proper spatio-temporal *Hox *regulation. Fine mapping of *Hox *interactions in other cell systems will help identify the DNA sequences and regulatory proteins mediating both conserved and cluster-specific contacts. In this study, we also developed valuable tools to identify CCSs of gene expression. These tools will be useful to identify leukemia *HoxA *CCSs and to assess the diagnosis and prognosis predictive value of this new type of signature. Finally, complete mapping of physical interaction networks during differentiation should help further understand how the underlying transcription network of cellular differentiation regulates gene expression. This study represents the initial step towards defining the very first high-resolution molecular picture of a physically networking genome *in vivo *during differentiation.

## Materials and methods

### Cell culture

THP-1 is a human myelomonocytic cell line derived from the peripheral blood of a 1-year-old infant male with acute monocytic leukemia. The THP-1 cell line was subcloned and one clone (THP-1.5) was selected for its ability to differentiate homogeneously in response to PMA (phorbol 12-myristate 13-acetate). The THP-1.5 clone was provided by the RIKEN Genome Exploration Research Group (Genome Sciences Center, RIKEN Yokohama Institute, Yokohama, Japan) and cultured in Roswell Park Memorial Institute medium (RPMI 1640; Invitrogen™, Burlington, ON, Canada) supplemented with 10% fetal bovine serum (HyClone, Logan, UT, USA). Medium also contained 50 μM 2-mercaptoethanol (Invitrogen™), 1 mM sodium pyruvate (Invitrogen™), 10 mM HEPES (Invitrogen™), and 1% penicillin-streptomycin (Invitrogen™) ('complete' RPMI). Cells were grown at 37°C in 5% CO_2 _atmosphere.

To induce cellular differentiation of THP-1, cells were grown in 225 cm^2 ^flasks to approximately 1 × 10^5 ^per 100 ml of complete RPMI. Twelve hours before differentiation, half volume of fresh media (50 ml) was added to each flask. For differentiation, cells were collected by centrifugation and resuspended at 2 × 10^5 ^per ml in complete RPMI containing 30 ng/ml PMA (Sigma^®^, St-Louis, MO, USA). THP-1 cells were incubated 96 hours in the presence of PMA or DMSO (control), and collected for RNA extraction and 3C library preparation.

### Real-time PCR quantification

Total THP-1 RNA was extracted from undifferentiated (DMSO control) and differentiated (PMA) cells with the GenElute™ Mammalian Total RNA Miniprep kit as recommended by the manufacturer (Sigma^®^). Reverse transcription was performed with oligo(dT)_20 _(Invitrogen™) using the Omniscript Transcription kit (Qiagen^®^, Mississauga, ON, Canada). Gene expression was quantified by real-time PCR with a LightCycler (Roche, Laval, QC, Canada) in the presence of SYBR Green I stain (Molecular Probes^®^, Burlington, ON, Canada). The RT-PCR primer sequences used in this analysis are summarized in Additional data file 1.

### Control 3C libraries

Control 3C libraries are used to correct differences in 3C primer pair efficiency. A control 3C library for the human *Hox *clusters was generated from BACs as previously described [12,45]. Briefly, an array of BAC clones covering the four *Hox *clusters and one gene desert region (ENCODE region ENr313 on chromosome 16) was mixed at equimolar ratio. Mixed BAC clones were digested with *Bgl*II and randomly ligated with T4 DNA ligase. The following BAC clones were used to generate the library: RP11-1132K14, CTD-2508F13, RP11-657H18, RP11-96B9, RP11-197K24. BAC clones were obtained from Invitrogen™.

### 3C analysis

Cellular 3C libraries were generated as previously described [12,45]. Briefly, undifferentiated (DMSO control) and differentiated (PMA) cells were fixed in the presence of 1% formaldehyde, digested with *Bgl*II and ligated under conditions promoting intermolecular ligation of cross-linked restriction fragments. 3C libraries were titrated by PCR with 3C primers measuring the IF of neighboring restriction fragments in the control gene desert region described above (see 'Control 3C libraries'). 3C library quality was verified by measuring the compaction of the gene desert control region as previously described. *HoxA *3C IFs were normalized by calculating the average log ratio of corresponding gene desert contacts in samples as previously described [12]. PCR conditions were described elsewhere [45]. At least three PCRs were performed for each interaction, and similar results were obtained from two different sets of 3C libraries. 3C PCR products were resolved on agarose gels containing 0.5 μg/ml ethidium bromide and visualized by UV transillumination at 302 nm. Gel documentation and quantification was performed using a ChemiDoc™ XRS system equipped with a 12-bit digital camera coupled to the Quantity One^® ^computer software (version 4.6.3; BioRad, Mississauga, ON, Canada). 3C primer sequences are presented in Additional data file 2.

### Generation of 5C libraries

Forward and reverse 5C primers were designed with the '5CPrimer' algorithm described below (see 'Informatics'). Multiplex 5C libraries were produced by mixing 58 alternating forward and reverse 5C primers corresponding to consecutive *Bgl*II fragments in the *HoxA *cluster and gene desert regions. This 5C experimental design yields 50% interaction coverage over both genomic regions and measures up to 841 possible contacts simultaneously.

5C library preparation was performed as previously described [16,25,45] with minor modifications. Briefly, 3C libraries were each mixed with salmon testis DNA (Sigma^®^) to a combined DNA mass of 1.5 μg, and with 3.4 fmol of each 5C primer in a final volume of 10 μl of annealing buffer (20 mM Tris-acetate pH 7.9, 50 mM potassium acetate, 10 mM magnesium acetate, and 1 mM dithiothreitol). Samples were denatured at 95°C for 5 minutes and annealed overnight at 48°C. Annealed samples were ligated with Taq DNA ligase (NEB, Ipswich, MA, USA) for 1 h at 48°C by adding 20 μl of ligation buffer containing 10 units of ligase (25 mM Tris-HCl pH 7.6, 31.25 mM potassium acetate, 12.5 mM magnesium acetate, 1.25 mM NAD, 12.5 mM dithiothreitol, 0.125% Triton X-100). Reactions were terminated by incubating samples 10 minutes at 65°C. 5C libraries were amplified by PCR with forward T7 (TAATACGACTCACTATAGCC) and reverse T3 primers (TATTAACCCTCACTAAAGGGA) as described previously. T7 and T3 primers are complementary to common 5' and 3' tail sequences of forward and reverse 5C primers, respectively. Unincorporated primers and other contaminants were removed from samples with the MinElute Reaction Cleanup kit as recommended by the manufacturer (Qiagen^®^). 5C primer sequences are summarized in Additional data file 3.

### Quality control of 5C libraries

Quantitative representation of chromatin contacts in 5C libraries was verified by measuring individual 5C products within amplified multiplexed 5C libraries. 5C products were amplified individually by PCR with specific internal primers, resolved on 2% agarose gels and visualized with ethidium bromide (0.5 μg/ml). Linear-range PCR detection was verified with two-fold serial dilutions of multiplex 5C libraries. Internal primer sequences are summarized in Additional data file 5.

### 5C library microarray analysis

Multiplex 5C libraries were prepared as described above (see 'Generation of 5C libraries') and amplified with forward T7 and reverse 5'-Cy3-labeled T3 PCR primers. Custom maskless arrays (NimbleGen Systems Inc., Madison, WI, USA) were designed with the '5CArray' computer program described below (see 'Informatics'). Each array featured the sense strand of all 46,494 possible 5C ligation products within and between the four human *Hox *clusters and gene desert region. The array contained several inter-region negative controls. Each feature was represented by 8 replicates of increasing length ranging from 30 to 48 nucleotides, which served to identify optimal feature length under our hybridization conditions. A detailed description of the array design is presented on our website (see the 'URLs' section below). Maskless array synthesis was carried out as previously described [46].

Hybridization was carried out with 50 ng of amplified Cy3-5C libraries and using the NimbleGen CGH Hybridization kit as recommended by the manufacturer and as previously described [47-49]. Arrays were scanned using a GenePix4000B scanner (Axon Instruments, Molecular Devices Corp., Sunnyvale, CA, USA) at 5 μm resolution. Data from scanned images were extracted using NimbleScan 2.4 extraction software (NimbleGen Systems, Inc.).

### Informatics

#### 5CPrimer

We developed a program named '5CPrimer' to design forward and reverse 5C primers directly from a given genomic region. The algorithm first scans a genomic region of interest supplied in FASTA format to identify the position of restriction sites for any enzyme selected. 5C primers are then designed iteratively starting from the center of each cut site. Single nucleotides corresponding to the genomic DNA sequence are added in a 3' to 5' direction. The melting temperature of the elongating primer is calculated after each addition using values from nearest-neighbor thermodynamic tables [50]. Nucleotides are added until an ideal melting temperature of 76°C is reached. Because 5C primer sequences are restricted by the position of cut sites, initial primer lengths are variable and may extend beyond maximum array feature lengths. To harmonize 5C library and array design, the length of 5C primers was restricted to 72 polymerization cycles, which corresponds to the optimal number during array synthesis. The number of polymerization cycles required to generate oligos on arrays is proportional to complexity, with low complexity oligos requiring more cycles and yielding shorter feature lengths. 5CPrimer also uses the RepeatMasker software to identify primers homologous to repeats or low-complexity genomic regions [51-54]. Such primers were previously found to generate false positives, and should be excluded from experimental designs. Resulting 5C primers contain genomic homology regions ranging from 19 to 37 bp in length. The 5CPrimer algorithm attaches a modified T7 universal sequence (TAATACGACTCACTATAGCC) at the 5' end of all forward primers, and a modified complementary T3 universal sequence (TCCCTTTAGTGAGGGTTAATA) to the 3' end of all reverse primers. Additionally, all reverse primers are phosphorylated on the 5' end. 5CPrimer output is a text file, which can be submitted directly for synthesis.

#### 5CArray

We developed a computer program named '5CArray' to design custom 5C microarrays for any genomic region(s) of interest. This program uses the output from the 5CPrimer algorithm to determine the sequence of array features, which correspond to any possible 5C products between the forward and reverse 5C primers used in a given study. In addition to full-length 5C products, the user can specify a range of feature lengths for each 5C product. Varying feature lengths are useful to identify the optimal hybridization conditions under defined experimental conditions. 5CArray typically designs eight oligos for each predicted 5C product. Oligo sizes are defined equally from the center of the reconstituted restriction site and include 30, 36, 38, 40, 42, 44, 46, and 48 nucleotide sequences (combined half-site feature lengths). Oligo sequences only include complementary genomic regions and always exclude T7 and T3 universal primer sequences. In cases where one of the 5C primers of the 5C product is short, the program simply stops adding nucleotides to that end of the oligo. 5CArray outputs each oligo to a text file with a unique ID code. If arrays are designed from several 5CPrimer files, the resulting text files need only be merged and can be directly submitted for array synthesis.

#### Interaction frequency calculation: the IF Calculator program

5C analysis was conducted with custom arrays featuring half-site probe lengths of 15, 18, 19, 20, 21, 22, 23 and 24 bp as described above (see '5CArray'). The 15-bp half-site probe signal is representative of background noise and is used to determine which of the remaining probe values should be included to calculate the average IF of its corresponding fragment pair. We developed the 'IF Calculator' program to automate exclusion of points close to background signal. For each interaction and starting from the longest half-site, IF Calculator first compares the signal of each probe to the value of the corresponding 15-bp probe. If a signal is found to be less than 150% of the 15-bp values, that half-site signal is discarded along with all remaining shorter probe length values. Corresponding 15-bp signals are then subtracted from the remaining values to remove background from each entry. Corrected values are used to calculate IFs by dividing cellular and BAC 5C signals of corresponding feature lengths. Interaction frequencies are finally averaged and the variance, count, and 95% confidence interval are reported in the final 5C dataset. If all probe length values are rejected as background, an IF value of zero is reported and is indicated as a missing data point.

#### Three-dimensional model prediction: the 5C3D program

The 5C3D program begins by converting the IFs to distances (D) as follows:



where IF(i, j) is the IF between points i and j and D(i, j) is the three-dimensional Euclidean distance between points i and j, (1 ≤ i, j ≤ N). Next, the program initializes a virtual three-dimensional DNA strand represented as a piecewise linear three-dimensional curve defined on N points distributed randomly in a cube. The program then follows a gradient descent approach to find the best conformation, aiming to minimize the misfit between the desired values in the distance matrix D and the actual Euclidean pairwise distance:



Each point is considered one-at-a-time and is moved in the inverse direction of the gradient ∇ of the misfit function (for which an analytical function is easily obtained), using a step size equal to δ*|∇|. Small values of δ (δ = 0.00005 was used) ensure convergence of the method but increase the number of iterations needed. The process of iteratively moving each point along the strand in order to decrease the misfit is repeated until convergence (change in misfit between successive iterations less than 0.001). The resulting set of points is then considered to be the best fit for the experimental data and is represented as a piecewise linear three-dimensional curve.

The width of the line is then modified to be proportional to the density of the number of base pairs in the genome per distance unit. This curve is then annotated with differently colored transparent spheres centered at the transcription start sites of the genes present along the DNA sequence. Another option is to surround the strand by identically colored transparent spheres having their vertices lying on the line to represent the uncertainty in the exact model of the DNA strand as well as to indicate the density of the number of base pairs in the genome per distance unit in the virtual representation.

#### Model comparison: the Microcosm program

In order to compare and find differences between any two models, we developed a program entitled 'Microcosm'. This program uses two 5C array datasets as input. Datasets feature the average IF values, variance, counts (or number of technical repeats), and 95% confidence intervals for each pair of points. To establish the robustness and significance of the observed structural differences, Microcosm selects an IF at random from the normal distribution of the corresponding mean and variance. This process is repeated for each fragment pair to generate 'randomly sampled' 5C array datasets based on original 5C data. Each randomly sampled dataset is then used individually by 5C3D to infer the best fitting model. The final models are next analyzed to determine the local density of the environment surrounding each gene *G*. The local density is defined as the total number of DNA base-pairs from any DNA segment that lies within the volume of a sphere of a fixed radius centered at *G*'s transcription start site. The process described above is repeated 100 times for each original 5C dataset to generate 100 individual models and local density estimates around each gene. The average local density, its variance and 95% confidence interval for the mean are then calculated for each gene and reported in a graphical format called a local density plot. Local density plots can be compared to identify genes with significant differences in local density. A *p*-value is calculated for each difference and corresponds to the probability of incorrectly predicting a difference in local densities assuming normality of the data. Small *p*-values therefore indicate strong degrees of confidence in the difference between the local densities of a gene's environment between two states. When correlated with corresponding changes in gene expression, these differences may indicate that transcription is regulated by changes in chromatin conformation.

### Databases

The May 2004 human reference sequence (NCBI Build 35) produced by the International Human Genome Sequencing Consortium was used for 3C experimental design (see 'URLs' section below).

### URLs

The human genome sequence is available at [55]. Detailed protocols and 3C/5C design support information can be found at [56]. Complete raw datasets and bioinformatics tools developed in this study are also available at [57]. Tools include '5CPrimer', '5CArray', 'IF Calculator', '5C3D', and 'Microcosm'.

## Abbreviations

3C: chromosome conformation capture; 5C: chromosome conformation capture carbon copy; BAC: bacterial artificial chromosome; CCS: chromatin conformation signature; IF: interaction frequency; PMA: phorbol myristate acetate.

## Authors' contributions

JF carried out the 3C and 5C experiments, quantified gene expression by real-time PCR, and developed the 5CPrimer and 5CArray programs. MR developed the IF Calculator, 5C3D and Microcosm computer programs. SS participated in the 3C and 5C experiments and the gene expression quantification by real-time PCR. MF designed and validated the real-time PCR gene expression quantification system and participated in the 3C experimental design. YH defined the cellular differentiation conditions, provided the cell system and the initial gene expression data. MB supervised and participated in the development of all computer programs. JD conceived the study, participated in its design and coordination, supervised the 3C, 5C and gene expression experiments, and drafted the manuscript. All authors read and approved the final manuscript.

## Additional data files

The following additional data are available with the online version of this paper. Additional data file 1 is a table listing the human primer sequences for quantitative RT-PCR analysis. Additional data file 2 is a table listing the human 3C primer sequences used in this study. Additional data file 3 is a table illustrating the 5C primer sequences generated with the 5Cprimer algorithm. Additional data file 4 is a figure illustrating quantitative detection of chromatin contacts in our 5C libraries. Additional data file 5 is a table listing the human internal 5C primer sequences for quality control of 5C libraries. Additional data file 6 is a figure demonstrating that 5C array results recapitulate 3C analysis. Additional data file 7 is a table listing the *p*-values of local chromatin densities around *HoxA *genes shown in Figure 8c.

## Supplementary Material

Additional data file 1 Human primer sequences for quantitative RT-PCR analysis. Click here for file

Additional data file 2 Human 3C primer sequences for *HoxA *and gene desert analysis of THP-1 libraries. Click here for file

Additional data file 3 5C primer sequences generated with the 5Cprimer algorithm. Click here for file

Additional data file 4 **(a) **Representative agarose gel resolution of amplified multiplex cellular and BAC 5C libraries. Libraries were generated by mixing 29 of each forward and reverse 5C primers with corresponding 3C libraries as described in Materials and methods. BAC 5C library products typically migrate more heterogeneously then cellular counterparts due to increased complexity. **(b) **Linear schematic representation of *HoxA *cluster region analyzed in (c). Cluster features are as described in Figure 2b. Predicted *Bgl*II restriction pattern below *HoxA *diagram is shown to scale and restriction fragment number is indicated below each line. Orange shading identifies 'fixed' 3C region and green boxes indicate position of interacting fragments. **(c) **Detection of individual 5C contacts in multiplex 5C libraries. Formation of four different 5C ligation products in cellular and BAC multiplex 5C libraries was measured with internal 5C primers (right). Interaction frequencies in undifferentiated and differentiated cells were expressed relative to neighboring 71 and 72 interaction, which was set at one. 5C internal priming results are compared to 3C results shown on the left. 3C data are from Figures 3 &4 except that interaction frequencies were expressed relative to contact neighboring fixed region as described above. Each histogram value represents the average of at least three PCR reactions and error bars correspond to standard error of the mean. 5C internal primer sequences are shown in Additional data file 4. Click here for file

Additional data file 5 Human internal 5C primer sequences for quality control of 5C libraries. Click here for file

Additional data file 6 **(a) **Diagram of the *HoxA *cluster region compared in (b, c). Features are as described in Figure 2b. Predicted *Bgl*II restriction pattern below *HoxA *schematic is shown to scale and restriction fragments are identified below each line. **(b) **3C chromatin interaction profiles from four different fixed cluster regions. Fixed fragment is indicated above each graph. 3C data are from Figures 3 &4 except that interaction frequency in each cellular state is expressed relative to contact neighboring fixed region, which was set at one. Each histogram value represents the average of at least three PCR reactions and error bars correspond to standard error of the mean. **(c) **5C chromatin interaction profiles from four different fixed cluster regions. 5C data are from Figures 5 &6 except that interaction frequency is presented as described in (b). Interaction frequencies are the average of at least three array technical repeats. Error bars represent standard error of the mean. Click here for file

Additional data file 7 *p*-values of local chromatin densities around *HoxA *genes. Click here for file

## References

[B1] Sell S (2005). Leukemia: stem cells, maturation arrest, and differentiation therapy.. Stem Cell Rev.

[B2] Rosenbauer F, Tenen DG (2007). Transcription factors in myeloid development: balancing differentiation with transformation.. Nat Rev Immunol.

[B3] Suzuki H, Forrest A, van Nimwegen E, Daub C, Balwierz P, Irvine K, Lassman T, Ravasi T, Hasegawa Y, de Hoon M, Katayama S, Schroder K, Carninci P, Akalin A, Ando Y, Arner E, Asada M, Asahara H, Bailey T, Bajic VB, Bauer D, Beckhouse A, Bertin N, Björkegren J, Brombacher F, Bulger E, Chalk AM, Chiba J, Cloonan N, The FANTOM Consortium (2009). The transcriptional network that controls growth arrest and differentiation in a human myeloid leukemia cell line.. Nat Genet.

[B4] Kleinjan DA, van Heyningen V (2005). Long-range control of gene expression: emerging mechanisms and disruption in disease.. Am J Hum Genet.

[B5] West AG, Fraser P (2005). Remote control of gene transcription.. Hum Mol Genet.

[B6] Berger SL (2007). The complex language of chromatin regulation during transcription.. Nature.

[B7] Heard E, Bickmore W (2007). The ins and outs of gene regulation and chromosome territory organisation.. Curr Opin Cell Biol.

[B8] Chambeyron S, Bickmore WA (2004). Does looping and clustering in the nucleus regulate gene expression?. Curr Opin Cell Biol.

[B9] Dekker J (2003). A closer look at long-range chromosomal interactions.. Trends Biochem Sci.

[B10] de Laat W, Grosveld F (2003). Spatial organization of gene expression: the active chromatin hub.. Chromosome Res.

[B11] Splinter E, Grosveld F, de Laat W (2004). 3C technology: analyzing the spatial organization of genomic loci *in vivo*.. Methods Enzymol.

[B12] Miele A, Gheldof N, Tabuchi TM, Dostie J, Dekker J, Ausubel FM, Brent R, Kingston RE, Moore DD, Seidman JG, Smith JA, Struhl K (2006). Mapping chromatin interactions by chromosome conformation capture (3C).. Current Protocols in Molecular Biology.

[B13] Tolhuis B, Palstra RJ, Splinter E, Grosveld F, de Laat W (2002). Looping and interaction between hypersensitive sites in the active beta-globin locus.. Mol Cell.

[B14] Palstra RJ, Tolhuis B, Splinter E, Nijmeijer R, Grosveld F, de Laat W (2003). The beta-globin nuclear compartment in development and erythroid differentiation.. Nat Genet.

[B15] Vakoc C, Letting DL, Gheldof N, Sawado T, Bender MA, Groudine M, Weiss MJ, Dekker J, Blobel GA (2005). Proximity among distant regulatory elements at the beta-globin locus requires GATA-1 and FOG-1.. Mol Cell.

[B16] Dostie J, Richmond TA, Arnaout RA, Selzer RR, Lee WL, Honan TA, Rubio ED, Krumm A, Lamb J, Nusbaum C, Green RD, Dekker J (2006). Chromosome conformation capture carbon copy (5C): a massively parallel solution for mapping interactions between genomic elements.. Genome Res.

[B17] Ling JQ, Li T, Hu JF, Vu TH, Chen HL, Qiu XW, Cherry AM, Hoffman AR (2006). CTCF mediates interchromosomal colocalization between Igf2/H19 and Wsb1/Nf1.. Science.

[B18] Murrell A, Heeson S, Reik W (2004). Interaction between differentially methylated regions partitions the imprinted genes Igf2 and H19 into parent-specific chromatin loops.. Nat Genet.

[B19] Liu Z, Garrard WT (2005). Long-range interactions between three transcriptional enhancers, active Vkappa gene promoters, and a 3' boundary sequence spanning 46 kilobases.. Mol Cell Biol.

[B20] Spilianakis CG, Lalioti MD, Town T, Lee GR, Flavell RA (2005). Interchromosomal associations between alternatively expressed loci.. Nature.

[B21] Spilianakis CG, Flavell RA (2004). Long-range intrachromosomal interactions in the T helper type 2 cytokine locus.. Nat Immunol.

[B22] Tsai CL, Rowntree RK, Cohen DE, Lee JT (2008). Higher order chromatin structure at the X-inactivation center via looping DNA.. Dev Biol.

[B23] Gavrilov AA, Razin SV (2008). Spatial configuration of the chicken alpha-globin gene domain: immature and active chromatin hubs.. Nucleic Acids Res.

[B24] Duan H, Xiang H, Ma L, Boxer LM (2008). Functional long-range interactions of the IgH 3' enhancers with the bcl-2 promoter region in t(14;18) lymphoma cells.. Oncogene.

[B25] Dostie J, Zhan Y, Dekker J (2007). Chromosome conformation capture carbon copy technology.. Curr Protoc Mol Biol.

[B26] Barendsen N, Mueller M, Chen B (1990). Inhibition of TPA-induced monocytic differentiation in THP-1 human monocytic leukemic cells by staurosporine, a potent protein kinase C inhibitor.. Leuk Res.

[B27] Tsuchiya S, Kobayashi Y, Goto Y, Okumura H, Nakae S, Konno T, Tada K (1982). Induction of maturation in cultured human monocytic leukemia cells by a phorbol diester.. Cancer Res.

[B28] Abrink M, Gobl AE, Huang R, Nilsson K, Hellman L (1994). Human cell lines U-937, THP-1 and Mono Mac 6 represent relatively immature cells of the monocyte-macrophage cell lineage.. Leukemia.

[B29] Iida S, Seto M, Yamamoto K, Komatsu H, Tojo A, Asano S, Kamada N, Ariyoshi Y, Takahashi T, Ueda R (1993). MLLT3 gene on 9p22 involved in t(9;11) leukemia encodes a serine/proline rich protein homologous to MLLT1 on 19p13.. Oncogene.

[B30] Swansbury GJ, Slater R, Bain BJ, Moorman AV, Secker-Walker LM (1998). Hematological malignancies with t(9;11)(p21-22;q23)--a laboratory and clinical study of 125 cases. European 11q23 Workshop participants.. Leukemia.

[B31] Ayton PM, Cleary ML (2003). Transformation of myeloid progenitors by MLL oncoproteins is dependent on Hoxa7 and Hoxa9.. Genes Dev.

[B32] Kroon E, Krosl J, Thorsteinsdottir U, Baban S, Buchberg AM, Sauvageau G (1998). Hoxa9 transforms primary bone marrow cells through specific collaboration with Meis1a but not Pbx1b.. EMBO J.

[B33] Thorsteinsdottir U, Sauvageau G, Hough MR, Dragowska W, Lansdorp PM, Lawrence HJ, Largman C, Humphries RK (1997). Overexpression of HOXA10 in murine hematopoietic cells perturbs both myeloid and lymphoid differentiation and leads to acute myeloid leukemia.. Mol Cell Biol.

[B34] Pession A, Martino V, Tonelli R, Beltramini C, Locatelli F, Biserni G, Franzoni M, Freccero F, Montemurro L, Pattacini L, Paolucci G (2003). MLL-AF9 oncogene expression affects cell growth but not terminal differentiation and is downregulated during monocyte-macrophage maturation in AML-M5 THP-1 cells.. Oncogene.

[B35] Biondi A, Cimino G, Pieters R, Pui CH (2000). Biological and therapeutic aspects of infant leukemia.. Blood.

[B36] Lewis EB (1978). A gene complex controlling segmentation in *Drosophila*.. Nature.

[B37] Krumlauf R (1994). Hox genes in vertebrate development.. Cell.

[B38] Duboule D, Morata G (1994). Colinearity and functional hierarchy among genes of the homeotic complexes.. Trends Genet.

[B39] Kmita M, Duboule D (2003). Organizing axes in time and space; 25 years of colinear tinkering.. Science.

[B40] Bickmore WA, Mahy NL, Chambeyron S (2004). Do higher-order chromatin structure and nuclear reorganization play a role in regulating Hox gene expression during development?. Cold Spring Harb Symp Quant Biol.

[B41] Morey C, Da Silva NR, Perry P, Bickmore WA (2007). Nuclear reorganization and chromatin decondensation are conserved, but distinct, mechanisms linked to Hox gene activation.. Development.

[B42] Chambeyron S, Bickmore WA (2004). Chromatin decondensation and nuclear reorganization of the HoxB locus upon induction of transcription.. Genes Dev.

[B43] Lanzuolo C, Roure V, Dekker J, Bantignies F, Orlando V (2007). Polycomb response elements mediate the formation of chromosome higher-order structures in the bithorax complex.. Nat Cell Biol.

[B44] Lanzuolo C, Roure V, Dekker J, Bantignies F, Orlando V (2007). Polycomb response elements mediate the formation of chromosome higher-order structures in the bithorax complex.. Nat Cell Biol.

[B45] Dostie J, Dekker J (2007). Mapping networks of physical interactions between genomic elements using 5C technology.. Nat Protoc.

[B46] Singh-Gasson S, Green RD, Yue Y, Nelson C, Blattner F, Sussman MR, Cerrina F (1999). Maskless fabrication of light-directed oligonucleotide microarrays using a digital micromirror array.. Nat Biotechnol.

[B47] Nuwaysir EF, Huang W, Albert TJ, Singh J, Nuwaysir K, Pitas A, Richmond T, Gorski T, Berg JP, Ballin J, McCormick M, Norton J, Pollock T, Sumwalt T, Butcher L, Porter D, Molla M, Hall C, Blattner F, Sussman MR, Wallace RL, Cerrina F, Green RD (2002). Gene expression analysis using oligonucleotide arrays produced by maskless photolithography.. Genome Res.

[B48] Kim TH, Barrera LO, Zheng M, Qu C, Singer MA, Richmond TA, Wu Y, Green RD, Ren B (2005). A high-resolution map of active promoters in the human genome.. Nature.

[B49] Selzer RR, Richmond TA, Pofahl NJ, Green RD, Eis PS, Nair P, Brothman AR, Stallings RL (2005). Analysis of chromosome breakpoints in neuroblastoma at sub-kilobase resolution using fine-tiling oligonucleotide array CGH.. Genes Chromosomes Cancer.

[B50] Breslauer KJ, Frank R, Blocker H, Marky LA (1986). Predicting DNA duplex stability from the base sequence.. Proc Natl Acad Sci USA.

[B51] RepeatMasker. http://www.repeatmasker.org/.

[B52] AB-*BLAST *(formerly WU-BLAST). http://www.advbiocomp.com/blast.html.

[B53] Benson G (1999). Tandem repeats finder: a program to analyze DNA sequences.. Nucleic Acids Res.

[B54] Jurka J, Kapitonov VV, Pavlicek A, Klonowski P, Kohany O, Walichiewicz J (2005). Repbase update, a database of eukaryotic repetitive elements.. Cytogenet Genome Res.

[B55] UCSC Genome Bioinformatics. http://genome.ucsc.edu/.

[B56] Dostie Lab. http://dostielab.biochem.mcgill.ca/.

[B57] Genome Network Platform. http://genomenetwork.nig.ac.jp/index_e.html.

